# EHMTI-0008. The international headache society classification of primary headaches is not supported by data and should be revised

**DOI:** 10.1186/1129-2377-15-S1-D57

**Published:** 2014-09-18

**Authors:** E Shevel, D Shevel

**Affiliations:** 1 R & D, Migraine Research Institute, Johannesburg, South Africa

## 

The International Headache Society classification of primary headaches in the International ClassificationI of Headache Disorders (ICHD) is almost universally accepted by researchers and clinicians. It is highly unlikely that reputable journals will accept submissions for publication if the cohorts have not been selected strictly according the ICHD. Likewise, in the clinical setting the appropriate treatment is prescribed according to how the patient’s headache is classified. But how reliable is the ICHD? As it does not appear to be based on data, its scientific validity is questionable. If indeed it cannot be scientifically validated, then the results of research based on the ICHD would be inaccurate. The practical implications of this are that the research data on the efficacy of migraine drugs is inaccurate. As a result, patients diagnosed according to this classification often receive the incorrect treatment.

No conflict of interest.

